# Validation of the robustness of blood-based biomarkers for predicting amyloid-β positivity in Chinese populations

**DOI:** 10.3389/fnagi.2025.1660755

**Published:** 2025-12-03

**Authors:** Ying Wang, Liang Cui, Feng-Feng Pan, Zhen Zhang, Lin Huang, Ya Miao, Yi-Hui Guan, Qi-Hao Guo

**Affiliations:** 1 Department of Gerontology, Shanghai Sixth People’s Hospital Affiliated to Shanghai Jiao Tong University School of Medicine, Shanghai, China; 2 Department of Nuclear Medicine & PET Center, Huashan Hospital, Fudan University, Shanghai, China

**Keywords:** Alzheimer’s disease, blood-based biomarkers, amyloid-β, p-tau217, biomarker robustness

## Abstract

**Background:**

Blood-based biomarkers (BBBs) of Alzheimer’s disease (AD) provide a promising, minimally invasive alternative for detecting cerebral amyloid-β (Aβ) pathology. However, a lack of robust validation across diverse platforms and populations has hindered their broader clinical adoption.

**Objective:**

This study aimed to cross-platform validation of the robustness of BBBs for predicting Aβ positivity in a Chinese population.

**Methods:**

The whole cohort (*N* = 1,254) of AD clinical spectrum underwent cognitive assessments, cranial MRI scans, and Aβ PET scans. Subcohort 1 (*N* = 504) underwent Simoa-based quantification of peripheral blood Aβ40, Aβ42, p-tau181, and NfL. Subcohort 2 (*N* = 262) underwent additional single molecule assays (Simoa) based quantification of p-tau217 and GFAP. The whole cohorts (Subcohort 1, Subcohort 2, and the remaining population) were measured for the aforementioned six biomarkers (Aβ40, Aβ42, p-tau181, p-tau217, NfL, and GFAP) using light-initiated chemiluminescent assays (LiCA). We validated the robustness of BBBs for predicting Aβ positivity in Chinese populations, with a focus on p-tau217.

**Results:**

The BBBs of Aβ42/40, p-tau181, p-tau217, GFAP, and NfL have demonstrated remarkable robustness in identifying Aβ positivity within the Chinese population, as evidenced by both LiCA and Simoa assays. Among these markers, p-tau217 has emerged as the most accurate, performing robustness in both the whole cohort and cognitively normal individuals. Utilizing a dual-threshold approach for p-tau217, only 16% of samples fell into the intermediate range, thus requiring additional Aβ PET testing.

**Conclusion:**

Blood-based biomarkers have demonstrated good robustness for predicting Aβ pathology in the Chinese population, with plasma p-tau217 standing out as the most promising marker for early detection of AD-related changes.

## Introduction

Dementia is a rapidly growing and increasingly complex global public health challenge, with two-thirds of affected individuals residing in low- and middle-income countries (Kalaria et al., 2024). China, with the largest population of dementia patients in the world (Jia et al., 2020), faces a particularly acute burden, as Alzheimer’s disease (AD) and other dementias is the fifth leading cause of death in the country (Ren et al., 2022; Yang et al., 2024). This places a significant strain on both public resources and healthcare systems. Early detection of AD is essential, as it can delay disease progression, reduce healthcare costs, and improve quality of life for patients.

The current diagnostic criteria for AD rely on the detection of brain amyloid-β (Aβ) through cerebrospinal fluid (CSF) analysis and Aβ positron emission tomography (PET) imaging, which are used to enhance diagnostic certainty (Jack et al., 2018). However, the widespread application of these biomarkers in clinical settings is severely limited by their invasiveness, high costs, and restricted availability (Fargo et al., 2016). As a result, there is an urgent need to validate and implement accessible, scalable, and cost-effective biomarkers that can be readily applied in clinical practice.

Blood-based biomarkers (BBBs) represent a promising alternative to Aβ PET and AD CSF biomarkers, offering a less invasive and more cost-effective approach to detecting AD pathology (Leuzy et al., 2022). Proposed AD BBBs include the blood Aβ42/40, phosphorylated tau (p-tau) species—such as p-tau181, p-tau217, and p-tau231—as well as non-specific biomarkers like glial fibrillary acidic protein (GFAP) and neurofilament light chain (NfL) (Jack et al., 2024). Advances in highly sensitive detection techniques, such as the single molecule assay (Simoa) and the Fujirebio lumipulse assay, have enabled accurate measurement of distinct brain-derived proteins in blood (Figdore et al., 2024b; Rissin et al., 2010). However, reported diagnostic accuracy remains inconsistent, with significant variability in the performance of plasma Aβ42/40 and p-tau assays across different platforms and manufacturers (Janelidze et al., 2021, 2023; Warmenhoven et al., 2025). This inconsistency has hindered widespread replication of these findings.

Over the past 3 years, large-scale Chinese cohorts have successively validated plasma p-tau217 and p-tau181 as robust indicators of amyloid-PET positivity. In a 2024–2025 nationwide study covering Shenzhen (GHABS, *N* = 425) and Shanghai (Huashan, *N* = 297), Lan et al. (2025) showed that p-tau217 (Simoa-ALZpath) classified Aβ-PET positivity with AUC 0.90–0.91 and outperformed p-tau181 (AUC 0.80–0.84) across the entire AD continuum; a two-cutoff strategy further reduced the indeterminate zone to 23%–28%, suggesting that ≈75% of scans could be avoided. Lin et al. (2025) reported the first East-Asian cross-cultural validation (Korea-Taiwan, *N* = 270) using identical Simoa assays; p-tau217 achieved AUC 0.936 for Aβ PET, with NPV 97.5% in the low-risk stratum and PPV 86% in the high-risk stratum, performances that were reciprocally confirmed when Taiwanese-derived models were tested in Korean participants.

In this study, we aimed to validate the robustness of BBBs for predicting Aβ positivity in Chinese populations. Our primary objective was to assess the performance of BBBs, including Aβ42/40, p-tau181, p-tau217, GFAP, and NfL, as detected by different platforms, in predicting Aβ positivity in two subgroups. We further validated the robustness of these biomarkers in a larger, full cohort and conducted robustness analyses in cognitively normal individuals to evaluate the inter-assay reproducibility with a particular focus on the potential of p-tau217 in predicting Aβ positivity. Additionally, by implementing a two-cutoff LiCA p-tau217 algorithm to minimize confirmatory PET/CSF testing in a large Shanghai memory-clinic and community sample.

## Materials and methods

### Participants

Between March 2019 and October 2024, a total of 1,254 individuals were recruited from the Shanghai Sixth People’s Hospital Affiliated to Shanghai Jiao Tong University School of Medicine through the outpatient memory clinic and community outreach efforts, as part of the Chinese Preclinical Alzheimer’s Disease Study (CPAS; Cui et al., 2023). Clinical diagnoses were subsequently established according to established criteria for cognitively unimpaired (CU) individuals, mild cognitive impairment (MCI), and AD dementia. CU participants were defined as those without cognitive impairment. MCI participants were diagnosed in accordance with the Petersen criteria (Petersen et al., 1997; Petersen, 2004), while the diagnosis of AD was made by experienced neurologists, following the National Institute on Aging and Alzheimer’s Association criteria for probable AD dementia (McKhann et al., 2011). A detailed description of the inclusion and exclusion criteria for participant recruitment is provided in our previous studies (Cui et al., 2023; Pan et al., 2022).

Complete datasets were obtained for all participants, including cognitive assessments, apolipoprotein E (APOE) genotyping (with APOE ε4 carriage defined as the presence of at least one ε4 allele), cranial MRI scans, Aβ PET scans and BBBs analyses. All tests, blood collections, and imaging procedures were completed within a 1-month period. Blood samples were collected from all participants using anticoagulant-containing vacutainers, centrifuged, aliquoted, and stored for blood biomarker analysis. All participants provided written informed consent after being fully informed of the study procedures and objectives.

### Clinical assessments

Each participant underwent a comprehensive cognitive assessment. The Chinese version of Addenbrooke’s Cognitive Examination III (ACE-III-CV) and the Chinese version of the Montreal Cognitive Assessment-Basic (MoCA-BC) were used to evaluate global cognitive function. An extensive neuropsychological battery was administered to assess three key cognitive domains: memory, language, and attention/executive function, as previously described (Cui et al., 2023). Specifically, memory function was assessed using the long-delayed recall and recognition components of the Auditory Verbal Learning Test (AVLT). Language ability was evaluated with the Animal Fluency Test (AFT, total score) and the 30-item Boston Naming Test (BNT, total score). Executive function was measured using parts A and B of the Shape Trail Test (STT, time to completion).

### Blood biomarker assessment

#### Light-initiated chemiluminescent assays (LiCA)

**FIGURE 1:**
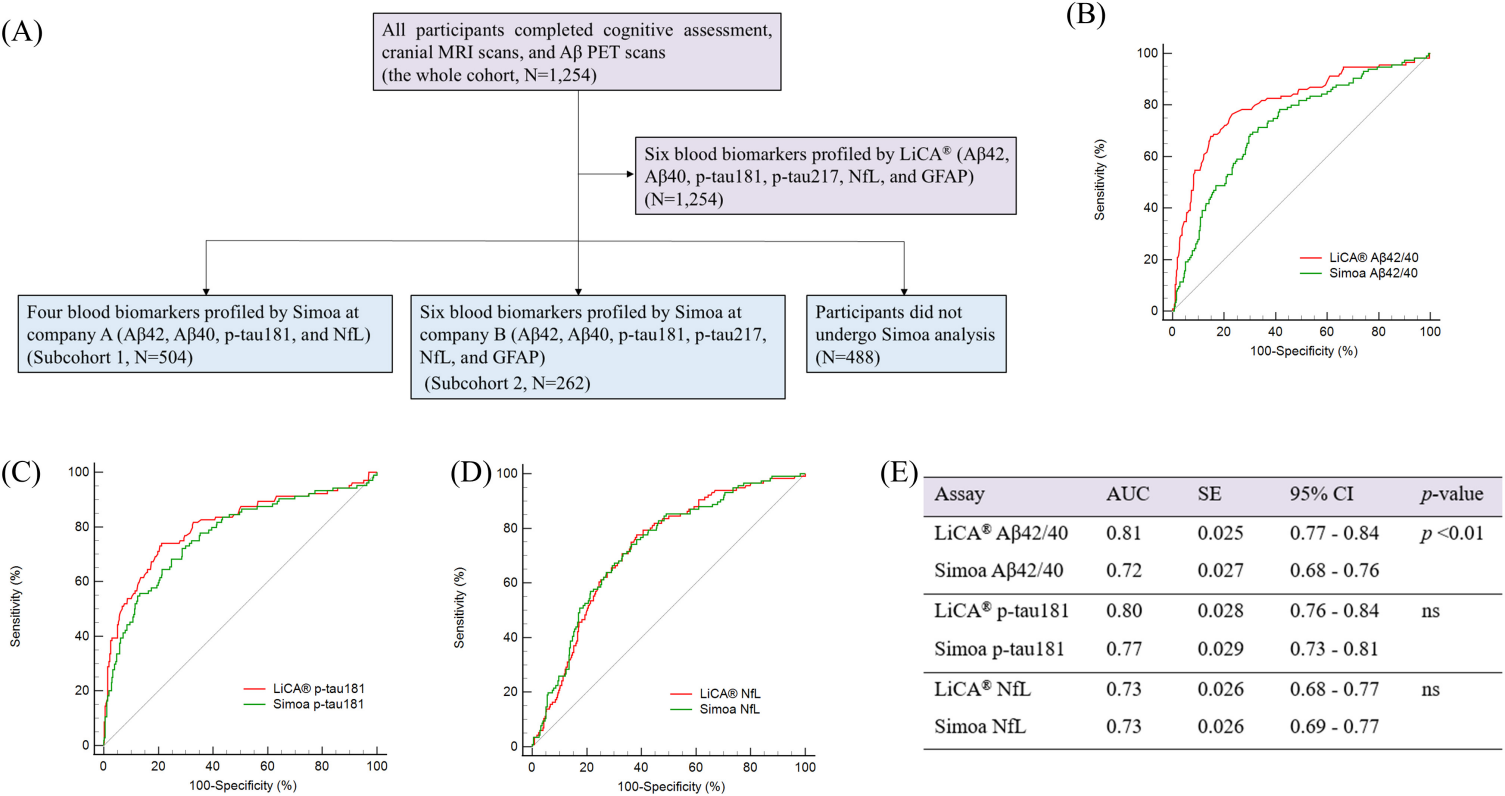
**(A)** Flowchart of the study population. **(B–E)** ROC curves illustrating the discriminative performance of LiCA versus Simoa assays for identifying abnormal Aβ PET in subcohort 1, with corresponding AUC values. ROC, receiver operating characteristic; AUC, area under the curve; Aβ, amyloid-β; PET, positron emission tomography; p-tau, phosphorylated tau; NfL, neurofilament light; ns, not significant. LiCA represents the Chemclin LiCA^®^ kits. Subcohort 1 [*N* = 504; mean age 67.8; Female, N (%) = 309 (61%)].

A total of 1,254 participants underwent blood testing for six biomarkers (Aβ42, Aβ40, p-tau181, p-tau217, GFAP, and NfL) using LiCA assays conducted by Chemclin Diagnostics, Beijing, China, in December 2024. These participants were collectively designated as the whole cohort, which was further stratified into two subcohorts (Figure 1A).

Testing was performed on the Chemclin LiCA 800 automated immunoassay analyzer (Chemclin Diagnostics, Beijing, China) using the Chemclin LiCA^®^ plasma Aβ42, Aβ40, p-tau181, p-tau217, NfL, and GFAP kits according to the manufacturer’s instructions (Ullman et al., 1996; Wang et al., 2022). EDTA-plasma samples were analyzed directly from the aliquot tubes without the need for cleanup, centrifugation, or dilution prior to measurement. LiCA employs a dual-antibody sandwich method with two-step incubations for blood biomarker detection. One antibody was pre-conjugated to nano-scale Chemibeads, while the other antibody was biotinylated. During the first step of incubation, target proteins within the sample form a sandwich complex by binding to both the Chemibead-immobilized detection antibody and the biotinylated capture antibody. In the subsequent step, streptavidin-coated Sensibeads are introduced, leveraging the high-affinity biotin-streptavidin interaction to bind the sandwich complex. Upon laser excitation, the Sensibeads generated singlet oxygen molecules that diffused into the Chemibeads, triggering a chemiluminescent reaction. In the absence of the target analyte, no sandwich complex is formed, resulting in unbound beads separated by a distance exceeding 200 nm. This spatial separation prevents singlet oxygen-mediated energy transfer, thereby eliminating background signal generation. The LiCA assays operates entirely in a homogeneous phase, eliminating the need for wash steps typically required in conventional immunoassay protocols. The principle of LiCA was illustrated in Supplementary Figure 1.

#### Quanterix Simoa assays

Within the whole cohort, 504 participants underwent additional testing for four AD blood biomarkers (Aβ40, Aβ42, p-tau181, and NfL) using the Simoa assay at Company A, forming subcohort 1. Additionally, 262 participants were tested for six AD blood biomarkers (Aβ40, Aβ42, p-tau181, p-tau217, GFAP, and NfL) using the Simoa assay at Company B, constituting subcohort 2. The remaining 488 participants did not undergo Simoa-based biomarker detection. The laboratory technicians were blinded to both the clinical diagnosis and the Aβ PET results.

The plasma biomarker testing was performed in Shanghai, China, by Company A and Company B using the Simoa platform (Quanterix, Billerica, MA, USA) according to the manufacturer’s instructions. At Company A, the Neurology 3-Plex A Assay Kit was used to measure Aβ42 and Aβ40, the P-tau 181 V2 Assay Kit was used for p-tau181, and the NF-light Assay Kit was used for NfL, as previously reported (Pan et al., 2023). At Company B, the Neurology 4-Plex E Advantage kit was employed to measure Aβ42, Aβ40, GFAP, and NfL, while the P-tau 181 V2 Assay Kit and the ALZpath p-Tau 217 V2 Assay Kit were used to measure p-tau181 and p-tau217, respectively, as described previously (Wu et al., 2024). The Simoa measurements for subcohorts 1 and 2 were both conducted on the Quanterix platform. All Simoa-based biomarker testing was completed within 1 year of blood collection. Subcohort 1 (*N* = 504) and Subcohort 2 (*N* = 262) were assembled by consecutive enrollment during March 2019–December 2021 and January 2023–January 2024, respectively. The assignment of all subcohorts was random. The measurement of p-tau217 and GFAP was not conducted in Subcohort 1 because the corresponding commercial assay kits were not available at the time of analysis. Consequently, Subcohort 2 was tested for six AD-related blood biomarkers following the commercial release of p-tau217 and GFAP kits.

### PET image acquisition and processing

Amyloid imaging was performed using [^18^F]florbetapir PET/CT scans on a Biography 64 PET/CT scanner (Siemens, Erlangen, Germany) at the PET Center of Huashan Hospital Affiliated to Fudan University. The imaging protocol followed well-established parameters as described in previous studies (He et al., 2024; Ren et al., 2023). Approximately 370 MBq ( ± 10%) of [^18^F]florbetapir was administered intravenously, followed by a 20-min PET scan acquisition after a 50-min uptake period. The acquired PET data were reconstructed using a filtered back-projection algorithm with comprehensive corrections for attenuation, normalization, dead time, photon attenuation, scatter, and random coincidences. The reconstructed PET images had a matrix size of 168 × 168 × 148 voxels, with a voxel dimension of 2.04 mm × 2.04 mm × 1.50 mm.

Using the Amyvid reading protocol, Aβ PET images were interpreted via visual inspection by three experienced raters who were blinded to the clinical diagnosis (He et al., 2024). Consensus among the raters determined the classification of participants as either Aβ positive (Aβ+) or Aβ negative (Aβ−) based on the PET images.

### Statistical analysis

Statistical analyses were conducted using SPSS version 23 (IBM SPSS) and MedCalc statistical software. For comparisons of characteristics between PET Aβ− and Aβ+ groups, independent-sample *t*-tests were used for normally distributed continuous variables, while Mann–Whitney U tests were applied for skewed continuous variables. Chi-square tests were used for categorical variables. Receiver operating characteristic (ROC) analysis was performed to evaluate the sensitivity and specificity of each blood-based biomarker in identifying individuals with abnormal Aβ PET status, with the area under the curve (AUC) calculated as a measure of diagnostic performance. Differences in AUC values among individual biomarkers were tested using the DeLong method. Optimal ROC cut points were determined based on the sensitivity and specificity.

Calculation method of dual cut point analysis: Only samples with values below the lower threshold were classified as negative and those above the upper threshold as positive; subsequent comparison with the PET gold standard yielded the Sensitivity (Sens)/Specificity (Spec). Intermediate values were not included in the calculation of Sens/Spec, but were incorporated into the gray zone proportion and the rate of requiring further examination.

## Results

### Participant characteristics by clinical and Aβ status

Table 1 summarizes the clinical characteristics and blood biomarker distribution of the participants. The study included 1254 participants, consisting of 554 CU individuals, 407 with MCI, and 293 with AD dementia, as determined by consensus clinical diagnosis. Across all groups, the proportion of females was 63% in the CU group, 60% in the MCI group, and 59% in the AD dementia group. The mean age of participants ranged from 65.2 years in the CU group to 69.2 years in the AD dementia group. The prevalence of both *APOE* ε4 carriage and Aβ positivity increased progressively with clinical severity. In the CU group, 19% were *APOE* ε4 carriers, compared to 29% in the MCI group and 47% in the AD dementia group. Similarly, the proportion of participants with abnormal Aβ PET were observed in 10% of the CU group, 28% of the MCI group, and 75% of the AD dementia group.

**TABLE 1:** The clinical characteristics, and blood biomarker distribution of participants.

Characteristic	CU (*N* = 554)	MCI (*N* = 407)	AD dementia (*N* = 293)	*p*-value
**Demographics**				
Age, years, mean (SD)	65.2 (7.3)	68.1 (7.0)	69.2 (8.3)	<0.001
Female, *n* (%)	348 (63)	244 (60)	174 (59)	0.529
Education, years, mean (SD)	12.7 (3.1)	11.2 (3.2)	9.3 (3.3)	<0.001
APOE ε4 carriers, *n* (%)	108 (19)	116 (29)	137 (47)	<0.001
Aβ PET positive, *n* (%)	53 (10)	112 (28)	221 (75)	<0.001
**Neuropsychological tests**				
ACE-III-CV, mean (SD)	81.8 (7.7)	70.6 (9.3)	49.0 (13.4)	<0.001
MoCA-BC, mean (SD)	25.5 (2.6)	21.1 (3.8)	12.7 (4.3)	<0.001
AVLT delayed recall, mean (SD)	5.3 (2.4)	2.0 (1.8)	0.51 (1.0)	<0.001
AVLT recognition, mean (SD)	21.8 (1.7)	18.3 (3.1)	15.7 (3.7)	<0.001
AFT, mean (SD)	17.7 (4.2)	13.3 (3.9)	10.1 (4.0)	<0.001
BNT, mean (SD)	24.6 (3.1)	21.4 (4.1)	18.2 (5.1)	<0.001
STT-A, mean (SD)	47.2 (15.3)	59.0 (24.0)	79.8 (39.6)	<0.001
STT-B, mean (SD)	127.6 (36.6)	168.2 (57.4)	205.7 (71.0)	<0.001
**LiCA assays**				
Aβ42/40, median (IQR)	0.030 (0.008)	0.028 (0.009)	0.024 (0.007)	<0.001
p-tau181, pg/mL, median (IQR)	4.79 (1.02)	5.00 (1.14)	6.02 (1.77)	<0.001
p-tau217, pg/mL, median (IQR)	0.33 (0.13)	0.36 (0.23)	1.13 (1.25)	<0.001
GFAP, pg/mL, median (IQR)	108.4 (53.5)	126.0 (81.4)	216.5 (155.8)	<0.001
NfL, pg/mL, median (IQR)	24.9 (11.1)	28.2 (11.3)	35.9 (14.2)	<0.001

LiCA represents the Chemclin LiCA^®^ kits. CU, cognitively unimpaired; MCI, mild cognitive impairment; AD, Alzheimer’s disease; Aβ, amyloid β; PET, positron emission tomography; APOE, apolipoprotein; ACE-III-CV, Chinese version of Addenbrooke’s Cognitive Examination III; MoCA-BC, Chinese version of Montreal Cognitive Assessment-Basic; AVLT, Auditory Verbal Learning Test; AFT, Animal Verbal Fluency Test; BNT, Boston Naming Test; STT-A and B, Shape Trail Test Part A and B; p-tau181, phosphorylated tau181; p-tau217, phosphorylated tau217; GFAP, glial fibrillary acidic protein; NfL, neurofilament light chain.

The participants were divided into two groups based on their Aβ PET status: Aβ negative and Aβ positive. Table 2 shows the demographic and clinical characteristics, as well as neuroimaging and plasma biomarker data. Significant differences were observed between the Aβ− and Aβ+ groups. Specifically, Aβ+ participants were older (*p* < 0.001 in subcohort 1 and the whole cohort; *p* < 0.01 in subcohort 2), had a higher prevalence of *APOE* ε4 carriers (all *p* < 0.001), and exhibited lower cognitive scores. Additionally, Aβ+ participants had lower education levels compared to Aβ− participants in subcohort 1 (*p* < 0.01) and the whole cohort (*p* < 0.001). In terms of plasma biomarkers, the Aβ+ group exhibited elevated levels of p-tau181, p-tau217, GFAP, and NfL, as well as a reduced Aβ42/40 ratio (all *p* < 0.001). No statistically significant differences were observed between the Aβ− and Aβ+ groups in terms of sex. Cognitive scale results are presented in Table 1: the Aβ+ group scored an average of 2.1 points lower on the MMSE, 2.7 points lower on the MoCA-BC, and 1.8 points lower on the AVLT delayed-recall subscore, all *p* < 0.001; the STT-B completion time was prolonged by 14 s, *p* < 0.001.

**TABLE 2:** The characteristics of the subcohort 1, subcohort 2, and the whole cohort.

Characteristic	Subcohort 1 (*N* = 504)			Subcohort 2 (*N* = 262)			The whole cohort (*N* = 1254)		
	Aβ− (*N* = 385)	Aβ+ (*N* = 119)	*p*-value	Aβ− (*N* = 177)	Aβ+ (*N* = 85)	*p*-value	Aβ− (*N* = 868)	Aβ+(*N* = 386)	*p*-value
**Demographics**									
Age, years, mean (SD)	65.1 (6.7)	68.0 (7.7)	<0.001	67.1 (7.9)	70.4 (7.7)	<0.01	66.0 (7.4)	69.7 (7.4)	<0.001
Female, *n* (%)	245 (64)	64 (54)	0.07	108 (61)	53 (62)	0.89	543 (63)	223 (58)	0.12
Education, years, mean (SD)	11.7 (3.0)	10.6 (3.6)	<0.01	12.6 (9.1)	11.6 (3.6)	0.34	11.9 (3.2)	10.8 (3.5)	<0.001
APOE *ε4* carriers, *n* (%)	62 (16)	57 (48)	<0.001	30 (17)	42 (49)	<0.001	155 (18)	206 (53)	<0.001
**Neuropsychological tests**									
ACE-III-CV, mean (SD)	76.4 (11.6)	60.2 (19.8)	<0.001	73.4 (14.2)	59.2 (17.2)	<0.001	75.6 (12.5)	59.2 (17.5)	<0.001
MoCA-BC, mean (SD)	23.7 (4.3)	17.5 (6.6)	<0.001	22.4 (5.1)	16.2 (5.5)	<0.001	23.3 (4.7)	16.4 (6.1)	<0.001
AVLT delayed recall, mean (SD)	4.4 (2.6)	2.1 (2.7)	<0.001	3.4 (2.4)	1.2 (2.0)	<0.001	4.1 (2.6)	1.5 (2.3)	<0.001
AVLT recognition, mean (SD)	20.7 (2.8)	18.2 (4.7)	<0.001	20.0 (2.8)	17.3 (3.7)	<0.001	20.5 (2.8)	17.5 (3.9)	<0.001
AFT, mean (SD)	15.7 (4.6)	13.9 (5.1)	<0.01	15.6 (5.2)	11.9 (4.8)	<0.001	15.6 (4.8)	12.5 (4.7)	<0.001
BNT, mean (SD)	22.2 (6.1)	15.8 (10.3)	<0.001	21.7 (6.1)	17.2 (9.0)	<0.001	23.2 (3.9)	21.0 (5.2)	<0.001
STT-A, mean (SD)	50.2 (19.2)	65.2 (38.8)	<0.01	52.7 (18.6)	67.7 (29.8)	<0.01	51.2 (18.7)	68.3 (35.4)	<0.001
STT-B, mean (SD)	134.2 (44.5)	161.6 (67.0)	<0.01	150.5 (54.0)	198.3 (62.0)	<0.001	141.3 (47.1)	183.4 (70.5)	<0.001
**Simoa assays**									
Aβ42/40, median (IQR)	0.053 (0.017)	0.044 (0.014)	<0.001	0.066 (0.020)	0.052 (0.011)	<0.001	–	–	
p-tau181, pg/mL, median (IQR)	1.59 (1.01)	3.02 (2.42)	<0.001	2.17 (1.38)	3.76 (1.68)	<0.001	–	–	
p-tau217, pg/mL, median (IQR)	–	–		0.28 (0.18)	1.02 (0.62)	<0.001	–	–	
GFAP, pg/mL, median (IQR)	–	–		43.7 (20.4)	85.4 (44.3)	<0.001	–	–	
NfL, pg/mL, median (IQR)	13.4 (7.7)	20.1 (9.4)	<0.001	15.4 (11.0)	22.7 (12.8)	<0.001	–	–	
**LiCA assays**									
Aβ42/40, median (IQR)	0.029 (0.007)	0.022 (0.005)	<0.001	0.032 (0.008)	0.023 (0.004)	<0.001	0.030 (0.008)	0.023 (0.005)	<0.001
p-tau181, pg/mL, median (IQR)	4.91 (1.11)	6.31 (2.23)	<0.001	4.67 (0.75)	6.05 (1.35)	<0.001	4.77 (0.96)	5.96 (1.56)	<0.001
p-tau217, pg/mL, median (IQR)	0.33 (0.15)	0.92 (1.01)	<0.001	0.33 (0.14)	1.22 (1.11)	<0.001	0.33 (0.13)	1.10 (0.97)	<0.001
GFAP, pg/mL, median (IQR)	105.5 (48.2)	162.0 (153.8)	<0.001	114.1 (61.5)	241.5 (149.3)	<0.001	109.1 (50.0)	218.5 (145.9)	<0.001
NfL, pg/mL, median (IQR)	22.9 (11.0)	29.9 (11.1)	<0.001	29.1 (11.5)	35.6 (14.4)	<0.001	25.8 (11.4)	33.6 (12.5)	<0.001

LiCA represents the Chemclin LiCA^®^ kits. Aβ, amyloid β; APOE, apolipoprotein; ACE-III-CV, Chinese version of Addenbrooke’s Cognitive Examination III; MoCA-BC, Chinese version of Montreal Cognitive Assessment-Basic; AVLT, Auditory Verbal Learning Test; AFT, Animal Verbal Fluency Test; BNT, Boston Naming Test; STT-A and B, Shape Trail Test Part A and B; p-tau181, phosphorylated tau181; p-tau217, phosphorylated tau217; GFAP, glial fibrillary acidic protein; NfL, neurofilament light chain.

### The performance of BBBs detected by different platforms in predicting Aβ positivity

We performed a comprehensive comparison of three BBBs (Aβ42/40, p-tau181, and NfL) measured by LiCA and Simoa assays to assess their predictive performance for Aβ status in subcohort 1. As illustrated in Figures 1B–E, ROC curves were generated to evaluate the performance of these biomarkers in detecting abnormal Aβ PET. The LiCA Aβ42/40 assay demonstrated significantly superior discriminative accuracy in identifying Aβ PET-positive individuals, achieving an AUC of 0.81 (95% CI: 0.77–0.84), compared to the Simoa Aβ42/40 assay, which yielded an AUC of 0.72 (95% CI: 0.68–0.76; *p* < 0.01). For p-tau181, the discriminative performance of the LiCA and Simoa assays was comparable, with AUCs of 0.80 (95% CI: 0.76–0.84) and 0.77 (95% CI: 0.73–0.81), respectively, showing no significant difference. Similarly, the LiCA and Simoa NfL assays exhibited equivalent performance, both achieving AUCs of 0.73 (95% CI: 0.68–0.77 for LiCA and 0.69–0.77 for Simoa). Notably, a comparison of p-tau217 and GFAP levels between the LiCA and Simoa assays was not conducted, as the Simoa assays were not utilized for the detection of these biomarkers in subcohort 1.

**FIGURE 2:**
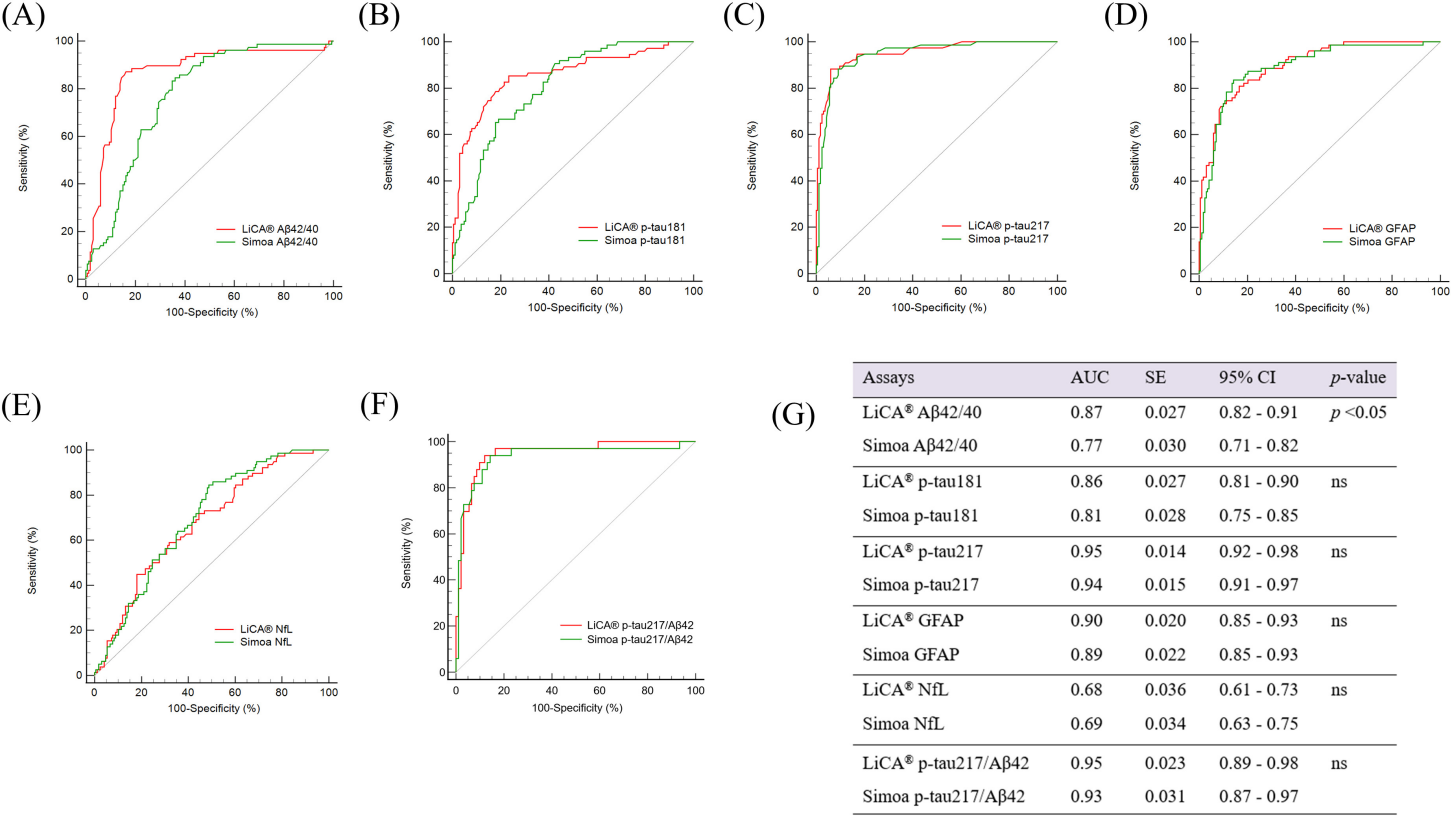
ROC curves illustrating the discriminative performance of LiCA versus Simoa assays for identifying abnormal Aβ PET in subcohort 2 **(A–F)**, with corresponding AUC values **(G)**. ROC, receiver operating characteristic; AUC, area under the curve; Aβ, amyloid-β; PET, positron emission tomography; p-tau, phosphorylated tau; GFAP, glial fibrillary acidic protein; NfL, neurofilament light; ns, not significant. LiCA represents the Chemclin LiCA^®^ kits. Subcohort 2 [*N* = 262; mean age 68.2; Female, N (%) = 161 (61.5%)].

Next, we performed a comparison of five BBBs—Aβ42/40, p-tau181, p-tau217, GFAP, and NfL—measured by LiCA and Simoa assays to evaluate their predictive performance for Aβ PET status in subcohort 2. Figure 2 displays the ROC curves for detecting abnormal Aβ PET in this subcohort using both assays. When identifying individuals with abnormal Aβ PET, the LiCA Aβ42/40 assay demonstrated significantly superior discriminative accuracy, achieving an AUC of 0.87 (95% CI: 0.82–0.91), compared to the Simoa Aβ42/40 assay, which yielded an AUC of 0.77 (95% CI: 0.71–0.82; *p* < 0.05). For p-tau181, the LiCA assay showed a higher AUC (0.86; 95% CI: 0.81–0.90) than the Simoa assay (0.81; 95% CI: 0.75–0.85), although this difference was not statistically significant. The discriminative performance of LiCA and Simoa assays for p-tau217 was comparable, with AUCs of 0.95 (95% CI: 0.92–0.98) and 0.94 (95% CI: 0.91–0.97), respectively. Similarly, the LiCA and Simoa GFAP assays exhibited equivalent performance, with AUCs of 0.90 (95% CI: 0.85–0.93) and 0.89 (95% CI: 0.85–0.93), respectively. Likewise, the LiCA and Simoa NfL assays demonstrated comparable performance, with AUCs of 0.68 (95% CI: 0.61–0.73) and 0.69 (95% CI: 0.63–0.75), respectively. The discriminative performance of LiCA and Simoa assays for p-tau217/Aβ42 was comparable, with AUCs of 0.95 (95% CI: 0.89–0.98) and 0.93 (95% CI: 0.87–0.97), respectively.

Blood-based biomarkers detected by different platforms have shown robustness in predicting Aβ positivity.

### Robustness analysis on the performance of BBBs in predicting Aβ positivity in the whole cohort

**FIGURE 3:**
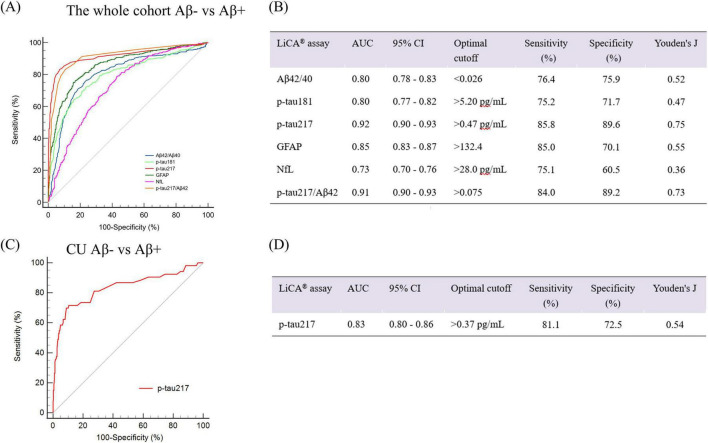
**(A,B)** ROC curves with Youden index cutpoints of blood-based biomarkers by LiCA for prediction of Aβ PET positivity in the whole cohort. **(C,D)** ROC curves with Youden index cutpoint of p-tau217 by LiCA for prediction of Aβ PET positivity in the CU individuals of the whole cohort. ROC, receiver operating characteristic; AUC, area under the curve; Aβ, amyloid-β; PET, positron emission tomography; p-tau, phosphorylated tau; GFAP, glial fibrillary acidic protein; NfL, neurofilament light; CU, cognitively unimpaired. LiCA represents the Chemclin LiCA^®^ kits.

To confirm the robustness of findings observed in the subcohorts, we further evaluated the diagnostic performance of BBBs measured by LiCA assays in differentiating Aβ status in the larger whole cohort. ROC curve analysis revealed that p-tau217 demonstrated the highest diagnostic accuracy for classifying Aβ PET status (Aβ+ vs. Aβ−), achieving an AUC of 0.92 (95% CI: 0.90–0.94). Among the biomarkers evaluated, p-tau217 emerged as the most accurate predictor of Aβ PET status, followed by p-tau217/Aβ42 and GFAP, which achieved AUCs of 0.91 (95% CI: 0.90–0.93) and 0.87 (95% CI: 0.84–0.89), respectively. Notably, Plasma p-tau217 levels exhibit significant differences among CU, MCI, and AD cohorts (Supplementary Figure 2). In contrast, p-tau181 and Aβ42/40 showed moderate diagnostic performance, with identical AUC values of 0.80 (95% CI: 0.77–0.82 for p-tau181 and 95% CI: 0.78–0.83 for Aβ42/40). NfL exhibited significantly lower diagnostic accuracy compared to the other biomarkers, with an AUC of 0.74 (95% CI: 0.71–0.76). The ROC curves for all biomarkers are illustrated in Figure 3A.

We have further established the cut-off values for these five biomarkers, among which p-tau217 has the highest sensitivity and specificity. An optimal cutoff value of 0.47 pg/mL for p-tau217 was established, yielding 85.8% sensitivity and 89.6% specificity in predicting Aβ status. The optimal cutoff values for all biomarkers are illustrated in Figure 3B. These findings highlight the superior performance of p-tau217 in predicting Aβ status.

### Diagnostic performance of p-tau217 in predicting Aβ positivity in CU individuals

Amyloid-β deposition exists even before the onset of clinical symptoms, and early identification of Aβ positive individuals allows for timely intervention or participation in clinical trials. Therefore, we further analyzed the diagnostic performance of plasma p-tau217 in CU individuals to validate the robustness of blood-based p-tau217 in identifying those with abnormal Aβ PET. Supplementary Table 1 summarizes the clinical characteristics and blood biomarker distribution of the participants. Compared to Aβ− participants, Aβ+ participants were older (*p* < 0.01), had a higher prevalence of *APOE* ε4 carriers (*p* < 0.001), exhibited lower cognitive scores in MoCA-BC (*p* < 0.05), and had elevated levels of p-tau181, p-tau217, GFAP, and NfL, as well as a reduced Aβ42/40 (*p* < 0.001 for Aβ42/40, p-tau181, p-tau217, GFAP, and *p* < 0.01 for NfL). No statistically significant differences were observed between the Aβ− and Aβ+ groups in terms of sex and education levels. As illustrated in Figure 3C, ROC curve analysis demonstrated that p-tau217 achieved an AUC of 0.83 (95% CI: 0.80–0.86) for identifying abnormal Aβ PET status in CU individuals. An optimal cutoff value of 0.37 pg/mL for p-tau217 was established, yielding 81.1% sensitivity and 72.5% specificity in predicting Aβ status (Figure 3D). As shown in Supplementary Figure 3, among other blood biomarkers, only p-tau 217/Aβ42 achieves AUC = 0.85 (comparable to p-tau 217 alone) in the CU population, while all other indicators yield AUC < 0.80.

The results indicate that p-tau217 demonstrates robust performance in predicting Aβ positivity among CU individuals.

### Diagnostic threshold determination (dual cutoffs) of LiCA p-tau217 for Aβ PET positivity

**FIGURE 4:**
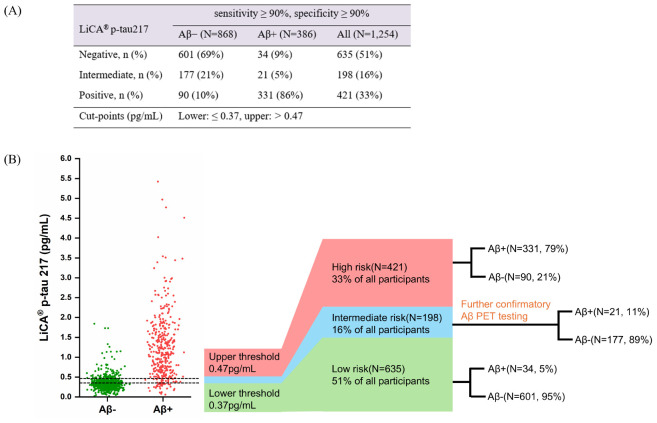
**(A)** LiCA p-tau217 assay performance for prediction of Aβ PET positivity at 90% sensitivity and 90% specificity cut-points in the whole cohort. **(B)** The clinical interpretation thresholds (dual cutoffs) for p-tau217 levels in the identification of abnormal Aβ PET status in the whole cohort. Aβ–, amyloid-β PET-negative; Aβ+, amyloid-β PET-positive; PET, positron emission tomography; p-tau, phosphorylated tau. LiCA represents the Chemclin LiCA^®^ kits.

Although LiCA-measured p-tau217 demonstrated significantly superior diagnostic performance compared to other LiCA-based biomarkers, it failed to achieve both sensitivity and specificity of ≥90% simultaneously using the single cutpoint method (Schindler et al., 2024). To enhance diagnostic precision and reduce false-positive outcomes relative to the single cutpoint method, we adopted a dual-threshold approach for the LiCA p-tau217 assay (Brum et al., 2023; Figdore et al., 2024a). Specifically, to achieve 90% sensitivity and specificity—thereby creating a practical clinical tool that minimizes the number of subjects falling into the intermediate “gray zone” (Sarto et al., 2023)—we established a lower cutoff value of p-tau217 ≤ 0.37 pg/mL and an upper cutoff value of p-tau217 > 0.47 pg/mL. Values below 0.37 pg/mL were deemed to have a high probability of being Aβ−, while those above 0.47 pg/mL were highly likely to be Aβ+. Figure 4A illustrates the distribution of LiCA p-tau217 stratified by Aβ status, with positive, negative, and intermediate “gray zones” demarcated by assay cutpoints corresponding to the 90% sensitivity and 90% specificity thresholds. Using this dual-threshold approach, 51% of all individuals had p-tau217 levels below the 0.37 pg/mL threshold, 33% had levels above the 0.47 pg/mL cutoff, and 16% fell into the intermediate range, requiring additional Aβ testing (CSF/Aβ PET) to confirm the presence of Aβ pathology (Figure 4B). In summary, by applying lower and upper cutoffs for plasma p-tau217, the Aβ status in 84% of all subjects could be predicted with reasonably high accuracy, potentially avoiding the need for more advanced biomarker testing (CSF/Aβ PET) in these cases.

Additionally, p-tau217 (dual cutoffs) measured by LiCA was found to perform well in predicting Aβ positivity across CU, MCI and AD participants, with gray-zone rates of 16.8%, 17.2% and 8.5% (Supplementary Table 2).

## Discussion

Alzheimer’s disease is a progressive neurodegenerative disorder that not only profoundly impacts patients and their families, but also imposes a significant socioeconomic burden on healthcare systems worldwide. China is rapidly entering an aging society, with the prevalence of AD on the rise. Early diagnosis by BBBs biomarker have thus become crucial strategies to mitigate its socioeconomic impact. This study validates the robustness of BBBs for predicting Aβ positivity in Chinese populations and confirms that p-tau217 is a promising blood biomarker to reduce diagnostic costs and enhance screening efficiency of AD.

Previous studies utilizing Simoa platform have established the diagnostic accuracy of BBBs for detecting Aβ pathology, with reported AUC ranges as follows: Aβ42/40 (0.69–0.78) (Janelidze et al., 2021), p-tau181 (0.76–0.88) (Karikari et al., 2020), p-tau217 (0.92–0.93) (Ashton et al., 2024), GFAP (0.80–0.82) (Arslan et al., 2025; Yang et al., 2023), and NfL (0.65–0.66) (Arslan et al., 2025; Yang et al., 2023). We performed a robustness comparison of blood biomarkers evaluated by LiCA and Simoa assays for predicting Aβ status in the Chinese population. Our findings indicated that plasma p-tau217, GFAP, p-tau181, and NfL detected by both Simoa and LiCA assays exhibited comparable efficiency in the entire AD spectrum in identifying individuals with Aβ PET positivity. Notably, the AUC for LiCA Aβ42/40 was significantly higher than that obtained from two central laboratories using different Simoa assay kits. This improved performance may be attributed to the LiCA platform’s streamlined workflow, which eliminates the need for sample dilution, thereby reducing potential adsorption or degradation of Aβ42 and Aβ40 during processing. The results revealed that plasma p-tau217, GFAP, p-tau181, Aβ42/40, and NfL detected by different platforms achieved robustness in predicting Aβ positivity within the clinical AD continuum. Among all BBBs analyzed by LiCA, p-tau217 emerged as the most accurate, with an optimal single cutoff value of 0.47 pg/mL yielding 85.8% sensitivity and 89.6% specificity in predicting Aβ status. This was followed by GFAP, p-tau181, and Aβ42/40, while NfL showed slightly inferior performance. Remarkably, p-tau217 achieved an AUC of 0.83 for identifying abnormal Aβ PET status in CU individuals, underscoring its potential as a reliable biomarker for the early detection of Aβ pathology during preclinical stages, even among CU individuals. Furthermore, the application of a dual-threshold combination for p-tau217 using LiCA revealed that only 16% of samples fell into the intermediate range, necessitating further Aβ PET testing to confirm Aβ pathology. This finding positions p-tau217 as an excellent candidate for distinguishing Aβ+ from Aβ− individuals as a single biomarker.

In our study, BBBs measured using Simoa exhibited performance in predicting Aβ PET positivity that was largely consistent with findings reported in the existing literature. Additionally, BBBs detected by different platforms (Simoa and LiCA) exhibited robustness in predicting Aβ positivity within two subcohorts comprising a total of 766 attendees, particularly highlighting p-tau217 as a robust discriminator of Aβ pathology (AUC in subcohort 2: LiCA 0.95 vs. Simoa 0.94). Furthermore, in the whole cohort of 1254 attendees and CU individuals, the pivotal role and robustness of p-tau217 measured by LiCA in detecting Aβ deposition was further confirmed. In addition, we validated the robustness on the performance of p-tau217 in predicting Aβ positivity in CU individuals. This real-world clinical setting enhances the translational potential of our findings by reflecting the actual diagnostic challenges encountered in routine practice.

In the present study, the AUC of the standalone biomarker p-tau217 on the LiCA platform was 0.92, which is consistent with findings from a contemporary Chinese cohort (Simoa-ALZpath p-tau217 AUC = 0.91) (Lin et al., 2025). We subsequently computed the p-tau217/Aβ42 ratio (*N* = 1,254), and its AUC was 0.91—slightly inferior to that of the standalone p-tau217 assay. This aligns with the findings of a 2025 meta-analysis published in Lancet Neurology: among 11 head-to-head comparison studies, the pooled AUC difference between standalone p-tau217 and the p-tau217/Aβ42 ratio was <0.02 (I^2^ = 0%). Thus, utilization of standalone p-tau217 in Chinese populations achieves discriminative efficacy comparable to that of the ratio-based model, while obviating the additional variability and costs inherent to Aβ42 quantification.

The dual cutoffs established for LiCA p-tau217 in this study (≤0.37 pg/mL for Aβ negativity, >0.47 pg/mL for Aβ positivity) categorized 16% of participants into the gray zone, reflecting an absolute 3% reduction relative to recent multicenter Simoa-ALZ path data (Lan et al., 2025), which reported a 19% gray zone. Currently, PET scans incur a relatively high cost. The application of the dual-threshold strategy enables 84% of subjects to forgo PET scanning, thereby achieving substantial cost savings. While Aβ PET remains the gold standard for detecting Aβ deposition, our preliminary research revealed that only 33.3% of Chinese cognitive clinic patients opt for this examination (Cui et al., 2023), with even greater implementation barriers in cognitively normal populations. P-tau217 holds substantial clinical promise by facilitating the early identification of at-risk subjects during the initial stages of pathological progression, thereby creating critical therapeutic windows for the timely implementation of disease-modifying interventions. Furthermore, plasma p-tau217 measured by LiCA could potentially serve as inclusion criteria (including enrichment and stratification) or be used to evaluate treatment efficacy in clinical trials for disease-modifying therapies (Teunissen et al., 2022). A compelling illustration of this biomarker-driven approach is demonstrated in the TRAILBLAZER-ALZ 2 trial (Donanemab, Eli Lilly), where p-tau217 was strategically integrated into the trial framework (Sims et al., 2023). This biomarker-stratified detection strategy not only offers significant economic advantages but also addresses the practical challenges currently impeding early screening practices for AD.

Our study also has several limitations. Firstly, although the participants represented a diverse patient population, the single-center design of this study, conducted in China, resulted in a racially homogeneous cohort. This limits the generalizability of our findings to other racial and ethnic groups. Secondly, the accuracy and true predictive power of the cutoffs derived from our findings need to be validated in diverse datasets with similar characteristics to ensure broader applicability and generalizability. Thirdly, the lack of longitudinal follow-up restricts the value of blood biomarkers in predicting disease progression. These limitations highlight the need for further multicenter studies involving diverse populations to strengthen the validity and generalizability of our conclusions.

## Conclusion

Our study demonstrates that, among the Chinese population, BBBs exhibit strong robustness in predicting Aβ positivity. Notably, plasma p-tau217 stands out as the most promising and superior biomarker for detecting AD pathology. Utilizing BBBs to determine Aβ positivity can enable more precise identification of early-stage patients, which holds significant importance for effectively addressing the growing burden of AD.

## Data Availability

The raw data supporting the conclusions of this article will be made available by the authors, without undue reservation.
