# Supplementary material for: Validation of the robustness of blood-based biomarkers for predicting amyloid-β positivity in Chinese populations
Source: Front Aging Neurosci. 2025 Dec 3;17:1660755. doi: 10.3389/fnagi.2025.1660755 (PMC12708543; doi:10.3389/fnagi.2025.1660755)
Supplement: Supplementary file 1 [file Data_Sheet_1.docx]

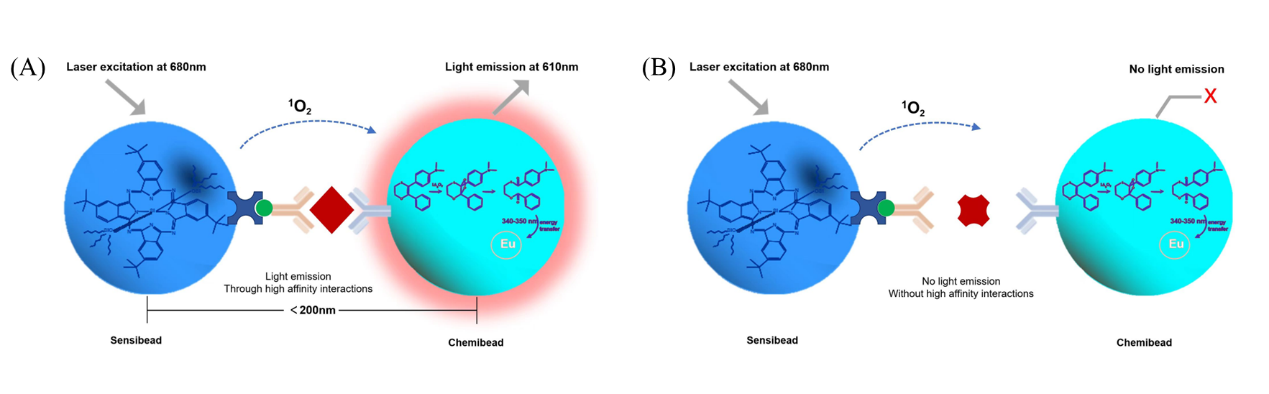


**Supplementary Figure 1 Principle of LiCA technology.** (A) Luminescence is induced in the presence of a specific substrate, (B) but fails to occur in its absence.

**Supplementary Figure 2** **Scatter plot of plasma p-tau217 by LiCA across CU, MCI, and AD groups.** p-tau, phosphorylated tau; CU, cognitively unimpaired; MCI, mild cognitive impairment; AD, Alzheimer's disease. LiCA^®^ represents the Chemclin LiCA^®^ kits. *p*<0.0001,****


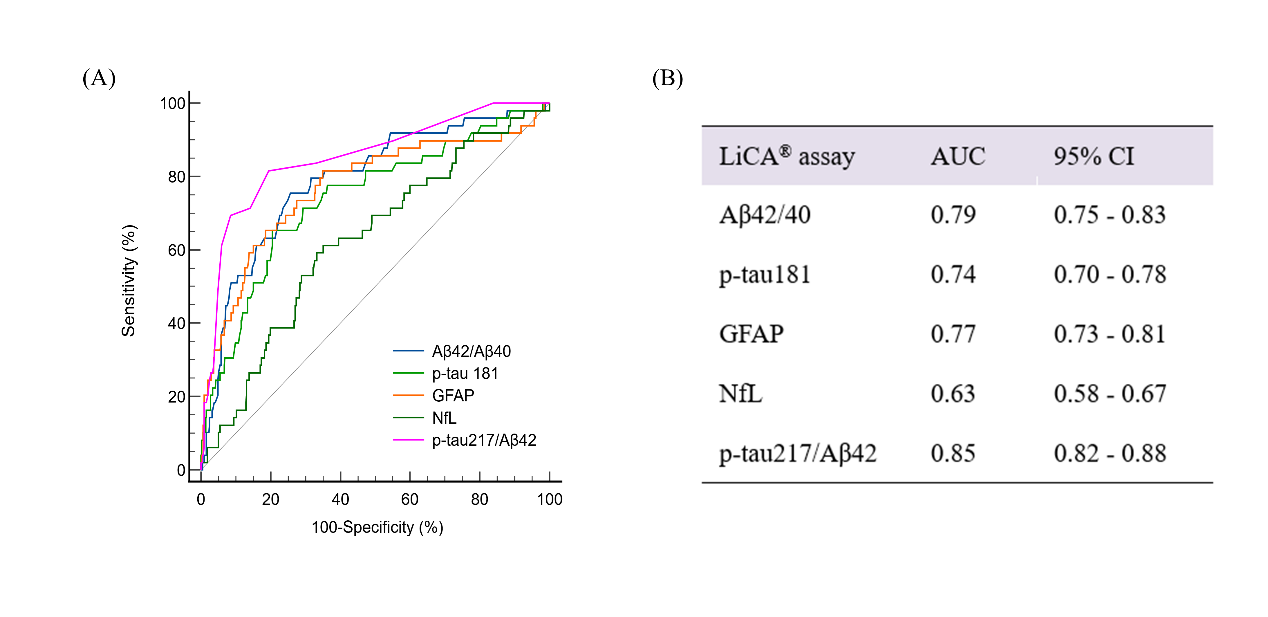


**Supplementary Figure 3 ROC curves of blood-based biomarkers by LiCA for prediction of Aβ PET positivity in the CU individuals of the whole cohort.** ROC, receiver operating characteristic; AUC, area under the curve; Aβ, amyloid-β; PET, positron emission tomography; p-tau, phosphorylated tau; GFAP, glial fibrillary acidic protein; NfL, neurofilament light; CU, cognitively unimpaired. LiCA^®^ represents the Chemclin LiCA^®^ kits.

**Supplementary Table 1 The characteristics of the CU participants in the whole cohort**

| **Characteristics** | **Aβ- (N=501)** | **Aβ+ (N=53)** | ***p*-value** |
| --- | --- | --- | --- |
| **Demographics** |  |  |  |
| Age, years, mean (SD) | 64.9 (7.3) | 68.1 (7.0) | <0.01 |
| Female, n (%) | 321 (64) | 27 (51) | 0.073 |
| Education, years, mean (SD) | 12.6 (3.1) | 12.8 (3.1) | 0.780 |
| APOE *ε4* carriers, n (%) | 84 (17) | 24 (45) | <0.001 |
| **Neuropsychological tests** |  |  |  |
| ACE-III-CV, mean (SD) | 81.8 (7.8) | 81.5 (7.5) | 0.78 |
| MoCA-BC, mean (SD) | 25.6 (2.5) | 24.6 (3.3) | <0.05 |
| AVLT delayed recall, mean (SD)  mean (SD) | 5.4 (2.3) | 4.6 (2.8) | <0.05 |
| AVLT recognition, mean (SD) | 21.8 (1.7) | 21.8 (1.6) | 0.80 |
| AFT, mean (SD) | 17.6 (4.1) | 18.2 (4.5) | 0.34 |
| BNT, mean (SD) | 24.6 (3.1) | 25.0 (2.9) | 0.32 |
| STT-A, mean (SD) | 47.0 (14.9) | 48.8 (19.2) | 0.51 |
| STT-B, mean (SD) | 126.4 (34.9) | 138.9 (49.2) | 0.08 |
| **LiCA^®^ assays** |  |  |  |
| Aβ42/40, median (IQR) | 0.030 (0.008) | 0.023 (0.006) | <0.001 |
| p-tau181, pg/mL, median (IQR) | 4.73 (0.98) | 5.50 (1.18) | <0.001 |
| p-tau217, pg/mL, median (IQR) | 0.32 (0.12) | 0.62 (0.51) | <0.001 |
| GFAP, pg/mL, median (IQR) | 105.0 (48.2) | 161.0 (125.4) | <0.001 |
| NfL, pg/mL, median (IQR) | 24.6 (11.1) | 29.5 (11.9) | <0.01 |

Note: LiCA^®^ represents the Chemclin LiCA^®^ kits.

Abbreviations: Aβ, amyloid β; APOE, apolipoprotein; ACE-III-CV, Chinese version of Addenbrooke’s Cognitive Examination III; MoCA-BC, Chinese version of Montreal Cognitive Assessment-Basic; AVLT, Auditory Verbal Learning Test; AFT, Animal Verbal Fluency Test; BNT, Boston Naming Test; STT-A and B, Shape Trail Test Part A and B; Aβ, amyloid-β; p-tau181, phosphorylated tau181; p-tau217, phosphorylated tau217; GFAP, glial fibrillary acidic protein; NfL, neurofilament light chain.

**Supplementary Table 2 The diagnostic efficacy of LiCA p-tau217 (dual cutoffs) across CU, MCI and AD groups**

| **Diagnostic status** | **Sensitivity (%)** | **Specificity (%)** | **Detection grey zone (%)** |
| --- | --- | --- | --- |
| **CU** | 81.1 | 89.8 | 16.8 |
| **MCI** | 86.6 | 90.9 | 17.2 |
| **AD** | 96.4 | 80.2 | 8.5 |

Abbreviations: p-tau, phosphorylated tau; CU, cognitively unimpaired; MCI, mild cognitive impairment; AD, Alzheimer's disease
